# Post-surgical Brachial Plexopathy

**DOI:** 10.7759/cureus.112109

**Published:** 2026-07-05

**Authors:** Hemant K Pandey, Kinsey J Gudenkauf, Joshua Lee

**Affiliations:** 1 Neurology, Brain and Spine Center, Chandler, USA; 2 Neurology, AT Still University KCOM (Kirksville College of Osteopathic Medicine), Mesa, USA; 3 Neurology, BASIS Chandler, Chandler, USA

**Keywords:** brachial plexopathy, brachial plexus injury, cervical discectomy, cervical spinal stenosis, denervation

## Abstract

Postoperative brachial plexopathy is a rare but recognized complication of cervical spine surgery, typically resulting from intraoperative traction, positioning, or compression of the brachial plexus. We present the case of a man in his 50s with poorly controlled diabetes who developed acute, severe upper-extremity pain, weakness, and numbness two days after a multilevel anterior cervical discectomy and fusion for cervical stenosis. Examination revealed C5-C6 deficits, and electromyography demonstrated active denervation in multiple muscles innervated by these roots. Magnetic resonance imaging (MRI) of the brachial plexus showed mild edema, while shoulder imaging revealed extensive rotator-cuff pathology. This case underscores the diagnostic challenge of distinguishing postoperative brachial plexopathy from simultaneous musculoskeletal disease and highlights the importance of early electrodiagnostic testing, careful imaging assessment, and timely management in high-risk patients.

## Introduction

Brachial plexopathy following cervical spine surgery is an uncommon occurrence but can have drastic consequences. The roots of the brachial plexus are located close to the operative field and are vulnerable to stretch, traction, and compression during surgery. It is reported that around 2.2% of people experience post-operative upper extremity palsy involving multiple segments after anterior decompression and fusion [1]. Risk factors, such as diabetes and prolonged operative time in certain positions, such as those with greater traction or nerve compression, further increase the likelihood of injury [2]. Typical clinical features of brachial plexopathy include an acute onset of severe upper extremity pain followed by weakness and sensory loss, consistent with how this patient presented. Clinical examination, electrodiagnostic testing, and magnetic resonance imaging (MRI) were utilized to differentiate brachial plexopathy from other types of nerve or shoulder injury, as it can be easily missed when multiple shoulder pathologies are present.

## Case presentation

A male in his 50s underwent cervical spinal surgery for cervical stenosis due to left upper extremity, neck, and chest pain with associated numbness and tingling in the arms bilaterally. He reported Lhermitte-like symptoms, described as an electric shock-like sensation that traveled down his arms when he flexed his neck. The surgery consisted of an anterior cervical diskectomy of C4-C5, C5-C6, C6-C7, and an interbody arthrodesis with fixation. Postoperatively, his preoperative symptoms initially resolved. Two days later, however, he awoke with severe proximal left arm pain and profound weakness. The pain radiated from the neck to the shoulder and below the elbow, was extreme at night, and had associated intermittent numbness and tingling to the fingertips and an inability to lift his arm. He also reported intermittent right-hand numbness without pain or weakness, occasional numbness of the feet, and chronic low-back pain radiating to the legs. The patient’s history included diabetes, hypertension, nephrolithiasis, prior right-shoulder surgery, and three lumbar discectomies at the L4-L5 level. He was a former heavy smoker, at six packs per day for 20 years, but quit around 20 years ago.

Physical examination revealed decreased pinprick sensation in the C5-C6 dermatomes, marked weakness of the left biceps, deltoid, supraspinatus, and infraspinatus, and scapular winging. Deltoid, biceps, and shoulder eversion/inversion muscle strength were 0/5. Wrist and finger flexion and extension were 4/5. There was left trapezius tenderness present, visible atrophy of the left upper extremity, decreased biceps and brachioradialis reflexes, and mild weakness graded at 4/5 for the right finger extensors and triceps.

Laboratory results revealed normal erythrocyte sedimentation rate, C-reactive protein, and total protein levels. Albumin, alpha-1-globulin, alpha-2-globulin, beta globulin, gamma globulin, and A/G ratio were also within normal limits. Immunoglobulins (IgG, IgA, and IgM) were within normal range, and antinuclear antibodies (ANA) screening was negative. The patient had an elevated hemoglobin A1c of 7.2% with an average estimated glucose of 160.

**Figure 1:**
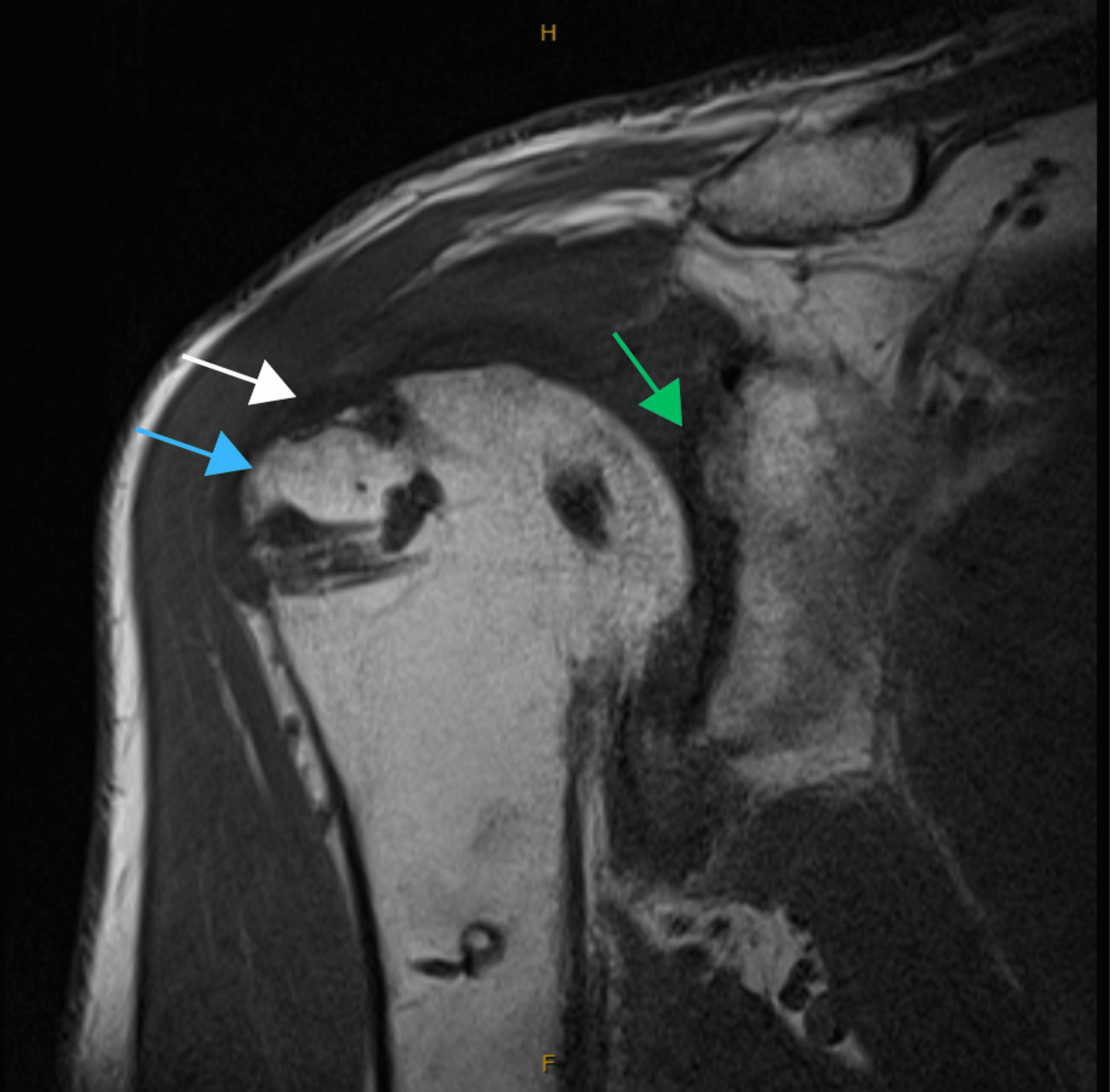
MRI of the left shoulder Coronal T1 MRI of the left shoulder with the white arrow indicating a high-grade partial thickness tear of the supraspinatus tendon, superimposed upon underlying tendinopathy and mild muscle atrophy. The blue arrow is displaying posterior subluxation of the greater tuberosity with respect to the glenoid. The green arrow indicates a degenerative type I and possibly type II superior labrum anterior to posterior tears.

**Figure 2:**
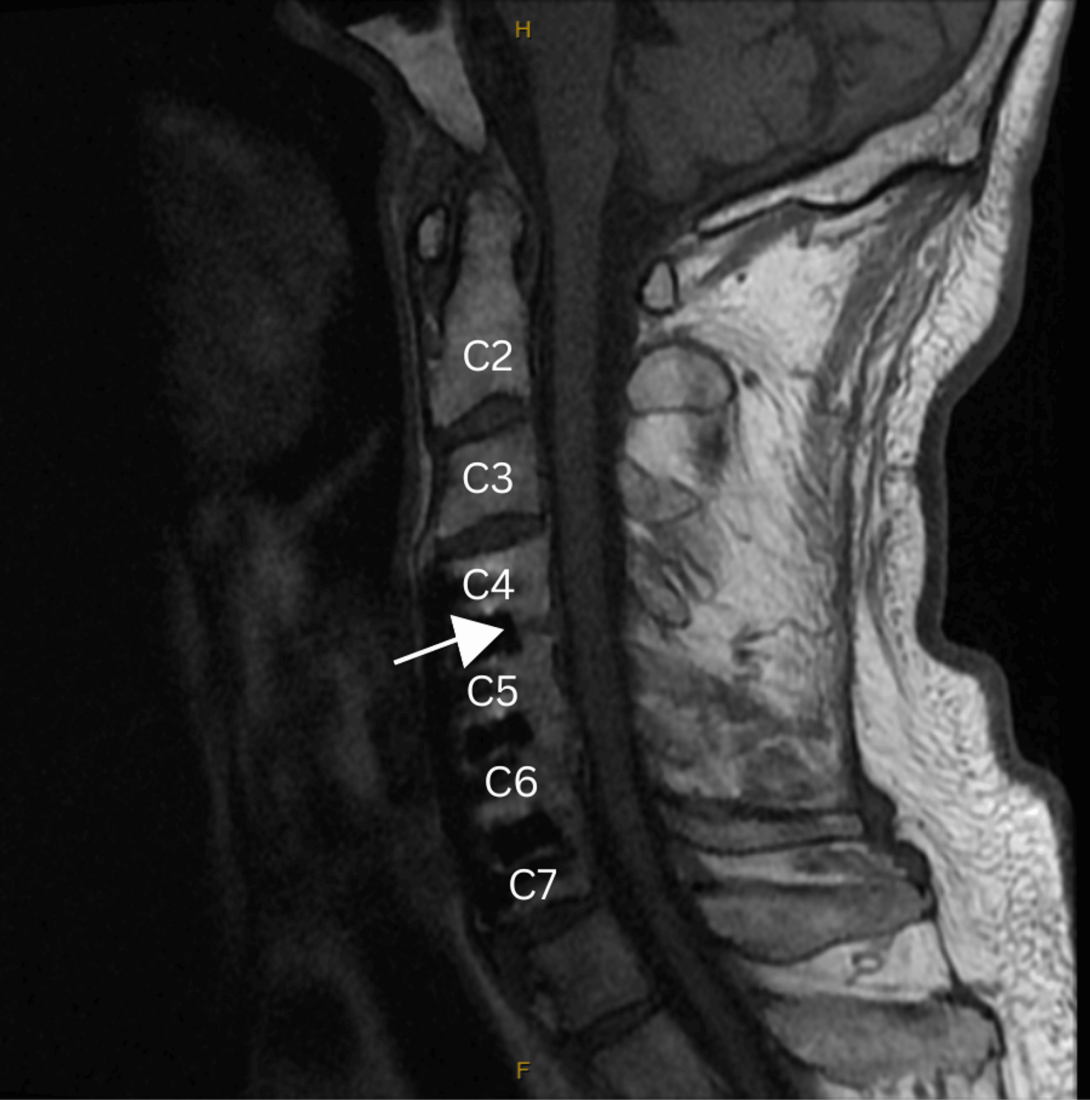
Cervical spine MRI Sagittal T1 MRI of the cervical spine demonstrating moderate to severe neural foraminal stenosis present at C4-C5 as indicated by the white arrow.

Nerve conduction studies were completed. The sensory nerve study showed evidence of left median and ulnar prolonged latencies (Table 1). A motor nerve study showed that the left median motor response had a slightly prolonged latency (Table 2). Needle electromyography (EMG) revealed severe denervation, ongoing and chronic, in the distribution of the left upper trunk of the brachial plexus (Table 3). Magnetic resonance imaging (MRI) of the shoulder showed a large area of tearing involving multiple muscles of the upper extremity. Findings included a high-grade, partial thickness, 17 x 24 mm articular surface and interstitial insertional tear of the supraspinatus tendon involving greater than 50-75% of tendon thickness, superimposed upon underlying tendinopathy and mild muscle atrophy (Figure 1). The infraspinatus tendon showed moderate to severe tendinopathy without any discrete tear and with mild muscle atrophy. The subscapularis tendon showed severe tendinopathy with a superimposed insertional delamination tear and mild to moderate muscle atrophy. There was also bicipital instability with flattening and superior medial subluxation of the biceps tendon, which likely reflected injury to the lateral biceps pulley sheath in the setting of anterior supraspinatus and superior subscapularis tendon tears. Additional findings included degenerative type I and possibly type II superior labrum anterior to posterior tears, posterior subluxation of the greater tuberosity with respect to the glenoid, mild arthrosis of the acromioclavicular joint, and type II curved acromion with lateral downsloping (Figure 1). MRI of the C spine showed neural foraminal stenosis that was moderate to severe on the left at C3-C4 and on the right at C4-C5 (Figure 2).

**Table 1:** Sensory nerve study

Stimulation Site	Latency (ms)	Peak Latency (ms)	Amplitude (μV)
Left Radial Nerve (Receptor Site: Wrist) 32°C			
Index	1.5	2.4	19.0
Left Palmar Nerve (Receptor Site: Middle Palm) 32°C			
Wrist	2.5	2.5	42.7
Ulnar Wrist	2.7	2.7	16.7

**Table 2:** Motor nerve study

Stimulation Site	Latency (ms)	Duration (ms)	Amplitude (mV)	Area (mVms)	Distance (mm)	Conduction Velocity (m/s)
Left Median Nerve (Receptor Site: Abductor Pollicis Brevis) 32°C						
Wrist	4.6	7.6	2.4	11.8	N/A	N/A
Elbow	9.6	7.8	2.3	10.9	275	55.0
Left Ulnar Nerve (Receptor Site: Abductor Digiti Minimi) 32°C						
Wrist	3.5	6.6	6.9	27.1	N/A	N/A
Elbow B	8.3	6.8	7.8	30.0	250	52.6
Elbow A	10.7	7.3	7.5	29.7	140	57.9

**Table 3:** Electromyography

Side	Muscle	Insertional Activity	Fibrillation Potentials	Positive Sharp Waves	Fasciculations	Polyphasic	Amplitude	Duration	Configuration	Pattern	Recruitment
Left	Deltoid	Increased	3++	3++	None	None	Normal	Normal	Normal	Normal	Decreased
Left	Biceps Brachii	Increased	3+	3+	None	Increased	Normal	Increased	Normal	Normal	Decreased
Left	Pronator Teres	Increased	1+	0	None	None	Normal	Normal	Normal	Normal	Normal
Left	Triceps	Normal	0	0	None	None	Normal	Normal	Normal	Normal	Normal
Left	Extensor Digiti Communis	Increased	2+	2+	None	None	Normal	Normal	Normal	Normal	Normal
Left	Dorsal Interosseous	Normal	0	0	None	None	Normal	Normal	Normal	Normal	Normal
Left	Infraspinatus	Increased	3+	3+	None	Increased	Normal	Normal	Normal	Normal	Decreased

A postoperative brachial plexopathy was the leading consideration, given the potential for intraoperative traction, positioning, or compression injury during cervical spine surgery. The clinical deficits localized to a C5-C6 distribution, consistent with a focal upper-trunk brachial plexus lesion. EMG confirmed denervation in multiple upper-extremity muscles innervated by these roots. The patient’s poorly controlled diabetes further increased the risk of a vascular insult, and the abrupt onset of symptoms strengthened the likelihood of a postoperative plexopathy.

Postoperative inflammatory neuropathy was also considered but ultimately ruled out. Inflammatory markers, including ESR and CRP, were within normal limits, immunoglobulin studies were unremarkable, and there were no systemic inflammatory signs or evidence of progressive, multifocal involvement that would suggest an immune-mediated etiology. A primary shoulder injury was another leading differential. MRI revealed multiple rotator-cuff tears and associated pathology that could contribute to pain and restricted range of motion. However, these structural lesions did not account for the dermatomal sensory loss, reflex depression, or the acute neurological presentation, making the shoulder injury more likely a comorbid condition rather than the primary cause. Diabetic peripheral neuropathy was also considered in light of the elevated hemoglobin A1c of 7.2%. This was excluded because the acute, focal onset and unilateral C5-C6 distribution were inconsistent with the symmetric, distal “stocking-glove” sensory loss typical of diabetic neuropathy. Finally, convulsive peripheral nerve injury was ruled out, as there was no history of seizures or other convulsive activity that could explain the presentation.

The final diagnosis was postoperative brachial plexopathy following multilevel anterior cervical discectomy and interbody arthrodesis with fixation. Neurologic examination revealed decreased pinprick sensation in the left C5-C6 dermatomes, marked atrophy and weakness of the left upper extremity, and reduced strength in the right triceps and finger extensors. The focal distribution of weakness and sensory loss, combined with the acute postoperative course, strongly supported brachial plexus involvement rather than an acute inflammatory neuropathy. EMG demonstrated significant active denervation across multiple muscles innervated by C5 and C6, including the deltoid, infraspinatus, biceps brachii, and pronator teres, further localizing the lesion to the plexus.

The patient was referred to an orthopedic surgeon who determined that operative intervention was not warranted and instead initiated a 4-week course of methylprednisolone sodium succinate. The patient began physical therapy and regularly used a transcutaneous electrical nerve stimulation (TENS) unit at home. At follow-up, he reported a moderate return of biceps flexion but no additional motor recovery.

Due to the patient's fasting blood glucose remaining elevated at approximately 250 mg/dL, his primary care physician started insulin therapy to improve glycemic control. At this stage, his neck pain was minimal, and the numbness and tingling in his left upper extremity had resolved, but he continued to have persistent pain in the left shoulder.

## Discussion

Following surgery of the cervical spine, it is critical to assess for postoperative brachial plexopathy. Diabetes and prolonged operative time are well-established risk factors that increase vulnerability to perioperative nerve injury, as demonstrated in this case. Here, diagnostic evaluation was further complicated by the presence of extensive structural shoulder pathology, which both mimicked and compounded the patient’s neurologic symptoms, underscoring the importance of careful differentiation between orthopedic and neuropathic causes of postoperative weakness and pain.

Electrodiagnostic studies remain essential for accurately localizing brachial plexus lesions and directing appropriate management [3]. Involvement of the C5-C6 roots, particularly affecting the deltoid and biceps, is commonly observed in brachial neuritis and was also evident in this patient [4]. The suprascapular, long thoracic, and axillary nerves are most frequently affected, and cervical spine surgery has been identified as a significant precipitating factor in similar cases [5]. Because injuries are usually regional, exhaustive testing of the entire plexus is usually not necessary [6].

Prompt recognition and timely treatment are necessary to limit symptom severity and prevent long-term disability. Early administration of high-dose intravenous corticosteroids, coupled with physical therapy and appropriate analgesia, is recommended to reduce inflammation and support functional recovery [7]. Timely intervention is particularly important because irreversible loss of neuromuscular end plates can occur within 20-24 months of denervation [4]. In this case, the patient received high-dose intravenous methylprednisolone and participated in structured physical therapy, which resulted in partial return of biceps flexion but persistent residual weakness, highlighting both the potential for recovery and the risk of lasting deficits despite appropriate management.

## Conclusions

Postoperative brachial plexopathy is a rare complication of cervical spine surgery that requires a high degree of clinical suspicion. It should be considered particularly in patients with established risk factors such as diabetes and multilevel procedures. Early localization through detailed neurologic examination and electrodiagnostic studies is critical in distinguishing a plexus-level injury from cervical radiculopathy or primary musculoskeletal disease. Adjunctive imaging, while helpful, must be interpreted cautiously in the presence of concurrent abnormalities that may confound the clinical picture. Timely initiation of treatment, including corticosteroid therapy, optimization of metabolic comorbidities, and structured rehabilitation, may improve functional outcomes; however, residual deficits can persist despite appropriate management. This underscores the importance of preventive strategies, including careful intraoperative positioning and minimization of traction on the brachial plexus.
